# *Jacaranda* flower (*Jacaranda mimosifolia*) as an alternative for antioxidant and antimicrobial use

**DOI:** 10.1016/j.heliyon.2020.e05802

**Published:** 2020-12-21

**Authors:** Humberto Aguirre-Becerra, Silvia Araceli Pineda-Nieto, Juan Fernando García-Trejo, Ramón G. Guevara-González, Ana Angelica Feregrino-Pérez, Beatriz Liliana Álvarez-Mayorga, Dulce María Rivera Pastrana

**Affiliations:** a Ingeniería en Biosistemas, Facultad de Ingeniería, Campus Amazcala, Universidad Autónoma de Querétaro, Chichimequillas-Amazcala Road Km 1 S/N, Amazcala, CP: 76265, El Marqués, Querétaro, Mexico; b Facultad de Química, Universidad Autónoma de Querétaro, Cerro de las Campanas S/N. Col. Las Campanas, CP: 76010, Santiago de Querétaro, Qro, Mexico

**Keywords:** *Jacaranda mimosifolia*, Antioxidant, Antimicrobial, Phenolic compounds

## Abstract

Antimicrobial resistance to antibiotics is a serious health problem worldwide, for this reason, the search for natural agents with antimicrobial power against pathogenic microorganisms is of current importance. The objective of this work was to evaluate the antioxidant capacity (ABTS+ and DPPH), antimicrobial activity, and polyphenol compounds of methanolic and aqueous extracts of *Jacaranda mimosifolia* flowers. The antimicrobial activity against *Bacillus cereus* ATCC 10876*, Bacillus subtilis* ATCC 6633*, Enterococcus faecalis* ATCC 51299*, Escherichia coli* ATCC 25922*, Listeria monocytogenes* ATCC 19115*, Pseudomonas aeruginosa* ATCC 27853*, Salmonella typhimurium* ATCC 14028, *Staphylococcus aureus* ATCC 25923, and *Streptococcus mutans* ATCC 25175, was determined using the Kirby Bauer technique. The results of polyphenolic compounds showed a high amount of total flavonoids in the methanolic and aqueous extracts (503.3 ± 86.5 and 245. 7 ± 27.8 mg Rutin Equivalents/g DW, respectively). Quercetin, gallic acid, caffeic acid, and rutin were identified by the HPLC-DAD technique, while in the GC-MS analysis, esters, fatty acids, organic compounds, as well as monosaccharides were identified. Higher antioxidant capacity was detected by the ABTS technique (94.9% and 62.6%) compared to DPPH values (52.5% and 52.7 %) for methanolic and aqueous extracts, respectively. The methanolic extract showed a greater inhibitory effect on gram-positive bacteria, with a predominant higher inhibition percentage on *Listeria monocytogenes* and *Streptococcus mutans* (86% for both). In conclusion, *Jacaranda* flower extracts could be a natural antimicrobial and antioxidant alternative due to the considerable amount of polyphenolic compounds, and serve as a sustainable alternative for the isolation of active ingredients that could help in agriculture, aquaculture, livestock, pharmaceutics, and other industrial sectors, to remediate problems such as oxidative stress and antimicrobial abuse.

## 1 Introduction

The abuse and inappropriate utilization of antibiotics to combat infectious diseases caused by microorganisms such as *S. aureus*, *E. faecalis*, *S. mutans*, *L. monocytogenes*, *P. aeruginosa*, and *E. coli* has generated an increase in antibiotic-resistant microorganisms and a diversification of resistance genes in bacteria (OMS, 2016). The immoderate use of these substances in humans, animals, and agriculture, combined with the not biodegradable characteristic of several of these compounds facilitate the transfer of resistant genes (Alós, 2015). Natural extracts are traditional components in drug development with a special use as natural preservatives that have raised as an alternative for antibiotic treatments. Some of their health-promoting properties are attributed to the presence of compounds with antimicrobial activities (Wei et al., 2013; Dai et al., 2020). The use of natural plant-derived antimicrobials can be highly effective in reducing the antibiotic dependence, minimizing the possibility of pathogen resistance to these components, and representing an alternative to cope with this global problem.

Diverse investigations have reported the antioxidant, antimicrobial, anticancer, and other biological properties of flowers and their extracts (Voon et al., 2012; Li et al., 2014; Al-Shukaili and Hossain., 2019; Karra et al., 2020). Moreover, flowers are common components in traditional medicine (Li et al., 2014) that can be gathered directly from the wild vegetation, allowing the population to take advantage of this resource (Magaña et al., 2010). *Jacaranda* is a member of the dicot family *Bignoniaceae Juss*., endemic plants of South America distributed in tropical areas throughout the world (Sánchez, 2011). Some species of this family are used in traditional medicine for the treatment of wounds, rheumatism, and colds. Additionally, its anti-malaria and antimicrobial activity have also been reported by Khamsan (2012). The objective of this work was to evaluate the antioxidant capacity (ABTS+ and DPPH), antimicrobial activity, and polyphenol compounds of methanolic and aqueous extracts of *Jacaranda mimosifolia* flowers. The extracts of this flower could be a potential sustainable alternative as an antimicrobial component and solution of oxidative stress problems in other organisms.

## 2 Materials and methods

### 2.1 Samples

Fresh flowers of *Jacaranda mimosifolia*, with no apparent physical, insect or microbial damage were collected in the months of March to May 2017 at the University Center of the Autonomous University of Querétaro, between the geographical coordinates ranging from 20° 35′ 33.7″ North latitude and 100° 24′ 44.5” West longitude. The flower petals were carefully removed (without anther, stamen or sepals) and were freeze-dried (freeze dryer Labconco FreeZone 4.5 L model 230 V, Kansas City, USA) for 48 h at -50 °C. Samples were ground, sieved (mesh size 30), sheltered from light, and stored until analysis at 4 °C.

### 2.2 Determination of total phenolic compounds

#### 2.2.1 Extracts preparation

Distilled water and methanol were used as solvents for the extraction of bioactive compounds. 50 mg of *Jacaranda* flower sample was weighed and mixed individually with 150 ml of solvent and kept under stirring in an orbital shaker (Lab Companion, Model SI 600R Bench top shaker) at 160 rpm for 24 h at room temperature and dark conditions (Cardador-Martínez et al., 2002). The methanol extracts were rota-evaporated and aqueous extracts were lyophilized.

#### 2.2.2 Determination of total phenolic content, tannins and total flavonoids

The Folin-Ciocalteu method (Singleton and Rossi, 1965), modified for use in 96-well microplate, was used for total phenolic content determination of flower extracts. The total phenolic content was expressed as mg Gallic Acid Equivalents (GAE)/g flower Dry Weight (DW). Condensed tannins were quantified at 492 nm (MULTISKAN GO, Thermo Fisher Scientific, Finland) and expressed in mg (+) Catechin Equivalents (CE)/g DW as described by Feregrino-Pérez et al. (2008). Total flavonoids determination was carried out using the methodology of Oomah (2005) where samples were measured at 404 nm and expressed as mg of Ruthin Equivalents/g DW.

### 2.3 Phytochemical profiling

#### 2.3.1 HPLC-DAD phenolic compound analysis

Analyses of phenolic compounds were performed on an Alliance 2695LC system (Waters) equipped with a 2998 diode array detector (DAD) set at 220–540 nm detection range, and a Symmetry C18 reverse phase column (100 × 4.6mm) (Waters). The eluent was a mixture of solvent A (water acidified with 0.1% formic acid) and solvent B (acetonitrile grade HPLC). A 30 μl volume of the phenolic extract was filtered through a 0.4 μm pore diameter syringe filter prior injection. Elution gradient was 5%–40% (B) from 0 to 18 min, 40%–90% (B) from 18 to 20 min and 90% (B) from 20 to 24 min at a flow rate of 0.5 mL/min and 35 °C. Individual compound identification and quantification was obtained by comparison of retention times and using a calibration curve of pure standard.

#### 2.3.2 Gas-chromatography-mass spectrometry (GC-MS) analysis

A GC–MS system (Agilent GC Series 7890A and Agilent single quadrupole MS detector model 5975C), with electron impact model (70 eV), a mass range of 50–700 m/z, HP-5MS capillary column (30 m × 0.25 mm i.d. x 0.25 μm), and splitless injection (at 250 °C) to 2.5 min/splitless time, was used for metabolite identification. The GC–MS control and data processing was performed by comparing their mass spectra using the Chem-Station (Agilent Technologies) software. Metabolite identification was analyzed through the spectral matching (MS) with commercialized libraries (NIST) based on the retention time, m/z, percentage of spectral similarity, and the internal standard.

### 2.4 Determination of antioxidant capacity

#### 2.4.1 DPPH method

DPPH antioxidant capacity (2,2-Diphenyl-1-picrylhydrazyl) was determined with the methodology described by Williams (1995) and modified by Fukumoto and Mazza (2000). The experiments were performed in triplicate. An absorbance of 520 nm was used at a time of 30 min. The results were expressed as the percentage of antiradical activity (*%ARA*) based on the following formula: *%ARA = [(A.Control)–(A.Sample)/A.Control]∗100* where *A.Control* represents the absorbance of the DPPH solution and *A.Sample* represents the absorbance of the sample with DPPH solution.

#### 2.4.2 ABTS method

ABTS (2,2′-azino-bis-(3-ethyl benzothiazolin-6-ammonium sulphonate)) assay was performed according to the method described by Nenadis et al. (2004). It was measured at 734 nm (MULTISKAN GO, Thermo Fisher Scientific, Finland), analyzing the samples in triplicate. The results were expressed as the percentage inhibition of ABTS based on the following formula: *%ABTS_inhibition = [(A.Control)-(A.Sample)/A.Control]∗100 where A.Control* represents the absorbance of the ABTS solution and *A.Sample* represents the absorbance of the sample with ABTS solution.

### 2.5 Antimicrobial activity

#### 2.5.1 Microorganism and growth conditions

Nine types of microorganisms were obtained from the collection of the Microbiology Laboratory, School of Chemistry, of the Autonomous University of Queretaro for the evaluation of the antibacterial activities of the flower extracts (methanol and aqueous). *Bacillus cereus* ATCC 10876*, Bacillus subtilis* ATCC 6633*, Enterococcus faecalis* ATCC 51299*, Listeria monocytogenes* ATCC 19115, *Staphylococcus aureus* ATCC 25923*, Streptococcus mutans* ATCC 25175 (gram-positive), *Escherichia coli* ATCC 25922*, Pseudomonas aeruginosa* ATCC 27853, and *Salmonella typhimurium* ATCC 14028 (gram-negative) were standardized to a concentration of 10^8^ CFU/mL for the antibacterial susceptibility test.

#### 2.5.2 Antimicrobial potential

Both methanol and aqueous extracts were used to evaluate the antimicrobial potential by using the modified agar disk diffusion method (Kirby et al., 1957; Washington, 1981) against a culture of pathogenic gram-positive and gram-negative bacterial strains. Four spots were uniformly placed inside the sensidisks: (1) methanol extract, (2) aqueous extract, (3) positive control (160 mg of Gentamicin), and (4) negative control (water or methanol).

Bacterial culture (100 μL) was inoculated into test tubes with molten Müller-Hinton agar at 45 °C. Afterward, the inoculation was placed into the plates according to the previously described arrangement. Six-diameter disks of sterilized Whatman filter paper were impregnated with 25 μL of the aqueous or methanol extracts (which corresponds to 20 and 145 equivalent mg of Gallic acid, for each extract, respectively), dried, and then, gently pressed on the inoculated agar plates. Subsequently, the plates were incubated at 37 °C for 24 h. The experiment was replicated three times. The inhibition halo was calculated by measuring the inhibition diameter and using the following formula of percent inhibition for each bacteria for each treatment: *%inhibition = [(Y-Z)/Y]∗100* where *Y* represents the growth of the bacteria without extract, *Z* represents the growth of bacteria with extract.

### 2.6 Statistical analysis

The results were presented as the mean ± standard error. Data were analyzed using a one-way ANOVA to determine significant differences. When differences were found, a mean comparison through a Tukey test was performed for the amount of polyphenolic compounds, antioxidant capacity, and the inhibition halo diameter between microorganisms. A Dunnet test was performed to find statistical difference between the methanol and aqueous extracts with respect to the positive control (Gentamicin).

## 3 Results and discussion

### 3.1 Determination of total phenolic content, tannins and total flavonoids

Plants have a secondary metabolism responsible for the biosynthesis of compounds called secondary metabolites which are involved in various non-essential processes, as stress adaptation, plant-to-plant communication, and pollinator attraction. Phenols, flavonoids, and condensed tannins, among other compounds, are examples of secondary metabolites. Phenols are organic compounds that contain at least one phenolic group and an aromatic ring attached to a hydroxyl group. Various biological activities, such as antioxidant, antibacterial, and antifungal, have been attributed to these specialized compounds (Li et al., 2016; Liu et al., 2016; Dai et al., 2020). The antimicrobial activity of phenolic compounds has been related to their ability to denature proteins and modify extracellular pH and hydrophobicity, considering them as surfactants (Büsra Konuk and Ergüden, 2020). The condensed tannin and flavonoids are the most important plant pigments for flower coloration. They are a subclass of polyphenols with an antioxidant, anti-inflammatory, antiplatelet, antiviral, antibacterial, vasodilator, as well as an antiallergic, and hepatoprotective properties, making these components a focus of scientific interest as possible adjuvants in treatments of a wide variety of diseases including those of microbial origin (Álvarez and Orallo, 2003). Additionally, flavonoids have inhibitory activity against diverse pathogens in plants, expanding their possible applications to the agricultural sector (Faggio et al., 2017).

*Jacaranda mimosifolia* flowers have a higher amount of total phenols, expressed as mg Gallic acid equivalent (GAE)/g DW (Table 1), compared to seed oil (0.02793 mg GAE/g) reported in Van-Nieuwenhove et al. (2019) and compared to the aqueous extracts of flowers reported by Rana et al. (2013) (49.80 mg GAE/g), both of the same species. Similarly, the results reported in the present research for total phenols are higher than those reported by Li et al. (2014) for 51 types of edible and wildflowers, with ranges from 0.13 to 11.48 mg GAE/g DW for the water-soluble and fat-soluble fractions, additionally, the total phenolic contents varied from 0.50 to 24.36 mg GAE/g DW. The same trend was observed for Jambu (*Acmella olerarea*) flowers, which are used in Amazon food and medicine, for both, in a dry and wet basis (5.65 ± 0.44 and 0.58 ± 0.04 mg GAE/g DW, respectively) (Andrade et al., 2018). Mak et al. (2013) showed lower results for ethanol and aqueous of hibiscus flower extracts (45.98 ± 1.07 and 54.36 ± 1.69 mg GAE/g DW, respectively), while for *Cassia* flower extracts (262.24 ± 4.50 and 94.68 ± 1.92 mg GAE/g DW, respectively) the results were superior compared to *J. mimosifolia* flowers (Table 1). The intrinsic (genetic, extracting solvent) and extrinsic (environmental, handling and development stage) factors have a strong influence on the production of phenolic compounds of plants (Fratianni et al., 2007).

**Table 1:** Quantification of polyphenolic compounds in the methanol and aqueous extracts of *Jacaranda mimosifolia* flowers.

	Total Phenols (mg GAE/g DW)	Condensed Tannin (mg CE/g DW)	Total Flavonoids (MG Ruthin/g DW)
Methanol extract	64.94 ± 10.45A	0.360 ± 0.098^B^	503.34 ± 86.55^A^
Aqueous extract	78.50 ± 4.76^A^	1.335 ± 0.096A	245.72 ± 27.88^B^

Superscript letters per column are found to be significantly different using ANOVA and the Tukey test at a significant level *p < 0.05.*

The amount of tannins differed significantly depending on the solvent medium, obtaining minor concentration for the methanol extract in comparison to the aqueous extract (Table 1). The concentration of tannins presented by the *Jacaranda mimosifolia* flowers is lower than those presented by other types of hibiscus flowers (2849.4 ± 121.1 and 4420.9 ± 110.7 mg CE/100 g DW), and *Cassia* flowers (1779.8 ± 139.2 and 103.3 ± 6.9 mg CE/100 g DW for ethanol and aqueous extracts, respectively) (Mak et al., 2013). The same trend is observed for *Limonium delicatulum* flowers for methanol and aqueous extracts (48.38 ± 0.70 and 8.14 ± 0.44 mg CE/g DW, respectively) (Medini et al., 2014), presenting higher values than these of flowers of *J. mimosifolia*. The presence of tannins can contribute to the elimination of free radicals depending on the number of aromatic rings, molecular weight, and nature of the hydroxyl group substitution (Cai et al., 2006).

The concentration of flavonoids is higher than the concentration of the other evaluated polyphenols (Table 1). The presence of flavonoids has been described in several investigations with extracts of flowers of the same species. Rana et al. (2013) reported 8.90 mg/g in the aqueous fraction of extracts with organic solvents, whereas Medini et al. (2014) reported 5.55 ± 2.95 and 2.15 ± 0.16 mg Ruthin/g DW for methanol and aqueous extracts *of L. delicatulum* flowers, respectively. Furthermore, it is observed that the amount of flavonoids is greatly influenced by the type of solvent medium used for the extraction. Flavonoids, the major group of phenolic compounds, contain a C6–C3–C6 skeleton and are by far the most diverse and common secondary metabolites in plants. Diverse modifications to the C3, such as hydroxylation, methylation, prenylation, and glycosylation, can occur to the baseline flavonoid molecules, producing a large number of flavonoids with diverse structures and different properties (Zha et al., 2019). Epidemiological studies indicate that flavonoids have health benefits (Gutiérrez-Venegas et al., 2019). The affection of cell membrane synthesis, nucleic acid inhibition, and metabolism of various bacterial strains provides flavonoids with bactericidal and bacteriostatic characteristics (Muhammad et al., 2019). However, the best-known property of flavonoids is the antioxidant effects, which are often much higher than those of vitamin E and vitamin C (Prior and Cao, 2000).

### 3.2 Phytochemical profiling

Metabolic profiling is a tool for screening the bioactive compounds and for the elucidation of regulative principles and pathways. Phenolic compounds are the base for the formation of different metabolites. Fatty acids and phytosterols are also related to the biosynthesis of metabolic pathways.

**Figure 1:**
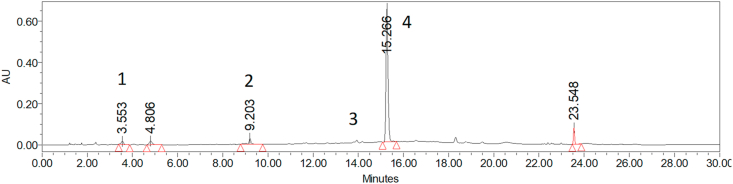
HPLC chromatograms of phenolic compounds identified in *Jacaranda mimosifolia* Flowers (220–540 nm): 1) gallic acid, 2) caffeic acid, 3) rutin and 4) quercetin.

For the HPLC analysis, gallic acid, caffeic acid, rutin, and quercetin (in elution order) were identified by comparison of the standard retention time and the UV spectra, and then quantified in the extract. Quercetin was the most abundant compound as shown in Figure 1 and Table 2. Diverse studies indicate that quercetin may have antioxidant, anti-inflammatory, anticancer, and gastroprotective activities (Bathori et al., 2004; Kanadaswami et al., 2005). Additionally, Li and Xu (2008), demonstrated the ability of some lotus leaf extracts with high concentration of quercetin to inhibit the growth of oral pathogenic bacteria. Rauha et al. (2000) reported antimicrobial effects of Finnish plant extracts containing phenolic compounds and other flavonoids as quercetin, quercetin-glycosides and derives.

**Table 2:** Compounds identified in *Jacaranda* flowers by HPLC-DAD.

Peak	Compound	RT (min)	λ max (nm)	mg/g DW of flower sample
1	Gallic acid	3.55	219, 27	6.35
2	Caffeic acid	9.20	240, 32	3.00
3	Rutin	13.93	254, 35	Traces
4	Quercetin	15.27	266, 34	83.5

For the GC-MS analysis, the screening of the *J. mimosifolia* extracts resulted in the presence of esters, organic compounds, fatty acids, monosaccharides, carotenoids and phytosterols. The mass spectrum range of the components was compared with the compound registry of the NIST and CAS library where 54 components were identified. The names of the recognized compounds, molecular weight, retention time, and concentration are presented in Table 3.

**Table 3:** Identified compounds in *Jacaranda* flowers by GC-MS.

No.	Compound	%∗	m/z	NIST/CAS	M.wt	RT
1	Silane, methoxytrimethyl	56.7	39	CAS: 1825-61-2	104	1.45 min
2	Acetamide, 2,2,2-trifluoro-N-(trimethylsilyl)-	71.5	119	CAS: 21149-38-2	257	2.40 min
3	Trisiloxane, octamethyl-	34.4	103	CAS: 107-51-7	236	2.60 min
4	Cystathionine, bis(trimethylsilyl) ester	29.4	215	CAS: 73090-79-6	366	3.27 min
5	L-Asparagine, N, N2-bis(tert-butyldimethylsilyl), tert-butyldimethylsilyl ester	10.4	156	CAS: 96381-41-8	474	4.34 min
6	Phosphoric acid, tris(trimethylsilyl) ester	97.6	76	CAS: 10497-05-9	314	5.17 min
7	Hydroquinone bis(trimethylsilyl) ether	58.6	167	CAS: 2117-24-0	254	6.84 min
8	[1,1′-Bicycloropropyl]-2-octanoic acid, 2′-hexyl-, methyl ester	52.7	155	CAS: 56687-	322	7.65 min
9	Butanedioic acid, [(trimethylsilyl)oxy]-, bis(trimethylsilyl)ester	88.6	67	CAS: 38166-11-9	350	8.34 min
10	L-Proline, 5-oxo-1-(trimethylsilyl)-, trimethylsilyl ester	79.3	83	CAS: 30274-77-2	273	8.97 min
11	Mannosamine	17	119	NIST: 127688	179	9.11 min
12	Benzoic acid, 3-[(trimethylsilyl)oxy]-, trimethylsilyl ester	21.3	94	CAS: 3782-84-1	282	9.55 min
13	α-D-Glucopyranoside, methyl 2-(acetylamino)-2-deoxy-3-O-(trimethylsilyl)-, cyclic methylboronate	16.9	236	CAS: 54477-01-9	331	10.63 min
14	Benzoic acid, 4-[(trimethylsilyl)oxy]-, trimethylsilyl ester	72.8	77	CAS: 27750-57-8	296	10.88 min
15	α-D-Glucopyranoside, methyl 2-(acetylamino)-2-deoxy-3-O-(trimethylsilyl)-, cyclic methylboronate	17.8	236	CAS: 54477-01-9	331	11.22 min
16	4-Trimethylsilyloxy-4-trimethylsilyloxycarbonylmethyl-2,5-cyclohexadiene-1-one	96.4	108	NIST: 280345	312	11.57 min
17	Pregn-5-en-20-one, 3-(acetyloxy)-16, 17-epoxy-6-methyl-, (3β, 16α)-	20.2	270	CAS: 55349-94-5	386	13.00 min
18	α-D-Galacotopyranose, 1,2,3-tris-O-(trimethylsilyl)-, cyclic methylboronate	12	228	CAS: 56196-95-3	420	13.31 min
19	Dihydroxyacetone dimer, tetra (trimethylsilyl)-	28.2	85	CAS: 28527-65-3	468	13.44 min
20	D-Psicofuranose, pentakis (trimethylsilyl) ether (isomer 2)	14.1	202	NIST: 380128	540	13.66 min
21	Glucopyranoside, methyl 2,3,5,6-tetrakis-O-(trimethylsilyl)-, α-D-	27.2	130	CAS: 6736-96-5	482	13.90 min
22	D-(-)-Fructofuranose, pentakis (trimethylsilyl) ether (isomer 1)	18.4	208	NIST:380166	540	14.11 min
23	L-(-)-Sorbofuranose, pentakis (trimethylsilyl) ether	11.2	168	NIST: 380164	540	14.25 min
24	D-(-)Ribofuranose, tetrakis (trimethylsilyl) ether (isomer 1)	14.3	148	NIST: 380115	438	14.28 min
25	D-Xylopyranose, 1,2,3,4-tetrakis-O-(trimethylsilyl)-	4.74	95	CAS: 55555-45-8	438	14.63 min
26	Glucofuranoside, methyl 2,3,5,6-tetrakis-O-(trimethylsilyl)-α-D-	21.7	130	CAS: 6736-96-5	482	14.73 min
27	1,5-Anhydro-D-sorbitol, tetrakis (triemethylsilyl) ether	21.7	230	NIST: 380138	452	15.11 min
28	1,2,3-Propanetricarboxylic acid, 2-[(trimethylsilyl)oxy]-,tris (trimethylsilyl) ester	13.4	70	CAS: 14330-97-3	480	15.22 min
29	Ribitol, 1,2,3,4,5-pentakis-O-(trimethylsilyl)-	15.9	185	CAS: 32381-53-6	512	15.33 min
30	β-D-(+)-Talopyranose, pentakis (trimethylsilyl) ether	6.08	151	NIST: 380171	540	15.57 min
31	14-Methyl-pentadecane1,2-diol, bis(trimethylsilyl) ether	24.4	132	NIST: 336515	402	16.12 min
32	Digitoxin	9.76	258	CAS: 71-63-6	764	16.69 min
33	Galactopyranose, 1,2,3,4,6-pentakis-O-(trimethylsilyl)-, β-d-	7.07	35	CAS: 1769-00-2	540	17.03 min
34	Hexdecanoic acid, trimethylsilyl ester	96.1	157	CAS: 55520-89-3	328	17.48 min
35	Tetradecane, 2,6,10-trimethyl-	16.9	112	CAS: 14905-56-7	240	18.22 min
36	Heptadecanoic acid, 10-methyl-, methyl ester	35.4	169	CAS: 2490-25-7	298	18.63 min
37	Silane, [(3,7,11,15-tetramethyl-2-hexadecenyl)oxy]trimethyl-	60.3	77	CAS: 57397-39-4	368	19.32 min
38	Imidazole, 2-fluoro-1-triacetylribofuranosyl-	16.4	232	NIST: 1292585	344	19.35 min
39	9,12-Octadecadienoic acid (Z,Z)-, trimethylsilyl ester	90.4	122	CAS: 56259-07-5	352	19.74 min
40	α-Linoleic acid, trimethylsilyl ester	84.8	127	CAS: 97844-13-8	350	19.84 min
41	Octadecanoic acid, trimethylsilyl ester	94.3	211	CAS: 18748-91-9	356	20.12 min
42	E-8-Methyl-9-1-ol-acetate	22.3	169	NIST: 130814	268	20.70 min
43	α-D-Glucopyranosiduronic acid, 3-(5-ethylhexahydro-2,4,6-trioxo-5-pyrimidinyl)-1,1-dimethylpropyl 2,3,4-tris-O-(trimethylsilyl)-, methyl ester	29.6	340	CAS: 55556-81-5	648	21.02 min
44	9-Octadecenamide, (Z)-	49.7	128	CAS: 301-02-0	281	21.62 min
45	Erucic acid	8.72	188	CAS: 112-86-7	338	23.21 min
46	9,12,15-Octadecanouc acid, 2-[(trimethylsilyl)oxy]-1-[[(trimethylsilyl)oxy]methyl]ethyl ester, (Z,Z,Z)-	44.2	189	CAS: 55521-23-8	496	23.561min
41	Estra-1,3,5 (10)trien-17β-ol	62.1	168	CAS: 2529-64-8	256	24.05 min
42	2-Monopalmitoylglycerol trimethylsilyl ether	57	229	CAS: 53212-97-8	474	24.44 min
43	Hexadecanoic acid, 2,3-bis [(trimethylsilyl)oxy]propyl ester	97.9	164	CAS: 1188-74-5	474	25.06 min
44	Docosanoic acid, trimethylsilyl ester	53.7	140	CAS: 74367-36-5	412	25.80min
45	1-Monolinoleoylglyccerol trimethylsilyl ether	58.4	230	CAS: 54284-45-6	498	27.94 min
46	2-Monostearin trimethylsilyl ether	77	161	CAS: 53336-13-3	502	28.74 min
47	1-Monolinoleoylglyccerol trimethylsilyl ether	77.6	230	CAS: 54284-45-6	498	28.92 min
48	9-Octadecenamide, (Z)-	55.3	124	CAS: 301-02-0	281	29.20 min
49	Octadecanoic acid, 2,3-bis [(trimethylsilyl)oxy]propyl ester	95.9	129	CAS: 1188-75-6	502	29.72 min
50	Squalene	61.1	96	CAS: 111-02-4	410	30.66 min
51	2-Monopalmitoylglycerol trimethylsilyl ether	58.1	229	CAS: 53212-97-8	474	32.77 min
52	Eicosane, 7-hexyl	20.3	179	CAS: 55333-99-8	366	33.03 min
53	Octadecanoic acid, 2,3-bis [(trimethylsilyl)oxy]propyl ester	33.2	313	CAS: 1188-75-6	502	34.27 min
54	(+)-α-Tocopherol, O-trimethylsilyl-	62.7	259	CAS: 2733-26-8	502	37.97 min

∗ The reported percentage corresponds to the percentage of similarity produced by the software library of the mass spectrometer.

Mostafa et al. (2014) identified different classes of phytochemicals as fatty acids, sterols and flavonoids in some *Jacaranda* species. In the present research, the most abundant identified compounds in the extracts of *J. mimosifolia* were esters, however, there is a considerable portion of fatty acids such as Erucid acid, which has been reported in the Brassicaceae family, specifically in *E. sativa*. Gulfraz et al. (2011) reported that the Erucic acid present in seeds of *E. sativa*, in both free and tryglyceride form, are responsible for the antimicrobial activity in Gram-negative and Gram-positive bacteria. In addition, there are also compounds of interest such as Digitoxin, Imidazole, and α-Tocopherol. Digitoxin is a cardiac glycoside with a potent antiviral and cancer cell growth inhibition activities (Cai et al., 2014). Imidazole is a heterocyclic ring and a structural part of some alkaloids that confers antifungal and antioxidant activity (Shalini et al., 2010) and α-Tocopherol is an important natural antioxidant with antiproliferative and protein kinase C-supressing effects (Engin 2009). Due to the diverse compounds found with the GC-MS analysis, the phenolic profile of the *Jacaranda* extract suggests that flavonoid quercetin in conjunction with esters, fatty acids, and other organic compounds could be responsible for the biological activities found in this study.

### 3.3 Determination of antioxidant capacity

The antioxidant activity in the methanol and aqueous extracts was evaluated with the most used methods to analyze antioxidant activity *in vitro, ABTS and DPPH* (Table 4). The methanol extract of *J. mimosifolia* flower showed a higher inhibition percentage on the ABTS radicals compared to the DPPH method with no significant difference between both extracts. These results are consistent with those reported by Rana et al. (2013) for aqueous extracts of flowers of the same species. The *Jacaranda* flower extracts presented a greater antioxidant effect, compared to other extracts of plants used within gastronomy and traditional medicine such as chia (*Salvia hispanica*) (2.4%–66.3% inhibition of ABTS and 4.7%–47.6% inhibition of DPPH) (Poungoué-Kaatou, 2006), saffron (15.7% inhibition of DPPH) (Gallo, 2010), and chamomile flower (14.5% inhibition of DPPH), taking into consideration that the difference in the inhibition percentage is also attributed to the used concentrations, type of extraction, environmental conditions and type of evaluated species (Salazar, 2009). A high concentration of polyphenols in the sample, mainly of the flavonoid type, contributes to greater efficiency in the elimination of free radicals. Mikolajczak et al. (2020) demonstrated a positive correlation (r = 0.75) between flavonoid content and antioxidant activity in various edible flowers. Thus, natural antioxidants can act as free radical scavengers, chain breakers, pro-oxidant metal ion complexes, and singlet-oxygen formation inhibitors (Tosun et al., 2011). On the other hand, recent studies have reported a greater amount of phenolic compounds, and therefore a greater antioxidant activity, in some flowers than other fruits and vegetables (De Morais et al., 2020). Phenolic compounds, in addition to their antioxidant capacity, have been proven a beneficial role as antimicrobial components and gene expression regulators involved in inflammation processes (Gallo, 2010).

**Table 4:** Determination of antioxidant capacity by ABTS and DPPH methods in methanol and aqueous extracts of *Jacaranda mimosifoli*a flowers.

	ABTS (% Inhibition)	DPPH (% ARA∗)
Methanol extract	94.91 ± 0.01^A^	52.51 ± 0.31^A^
Aqueous extract	62.63 ± 0.06^B^	52.77 ± 0.01^A^

∗ ARA = anti-radical activity. Extract concentration: 1 g DW/10 mL of solvent. Superscript letters per column are found to be significantly different using Tukey test at a significant level *p < 0.05*.

### 3.4 Antimicrobial activity

**Figure 2:**
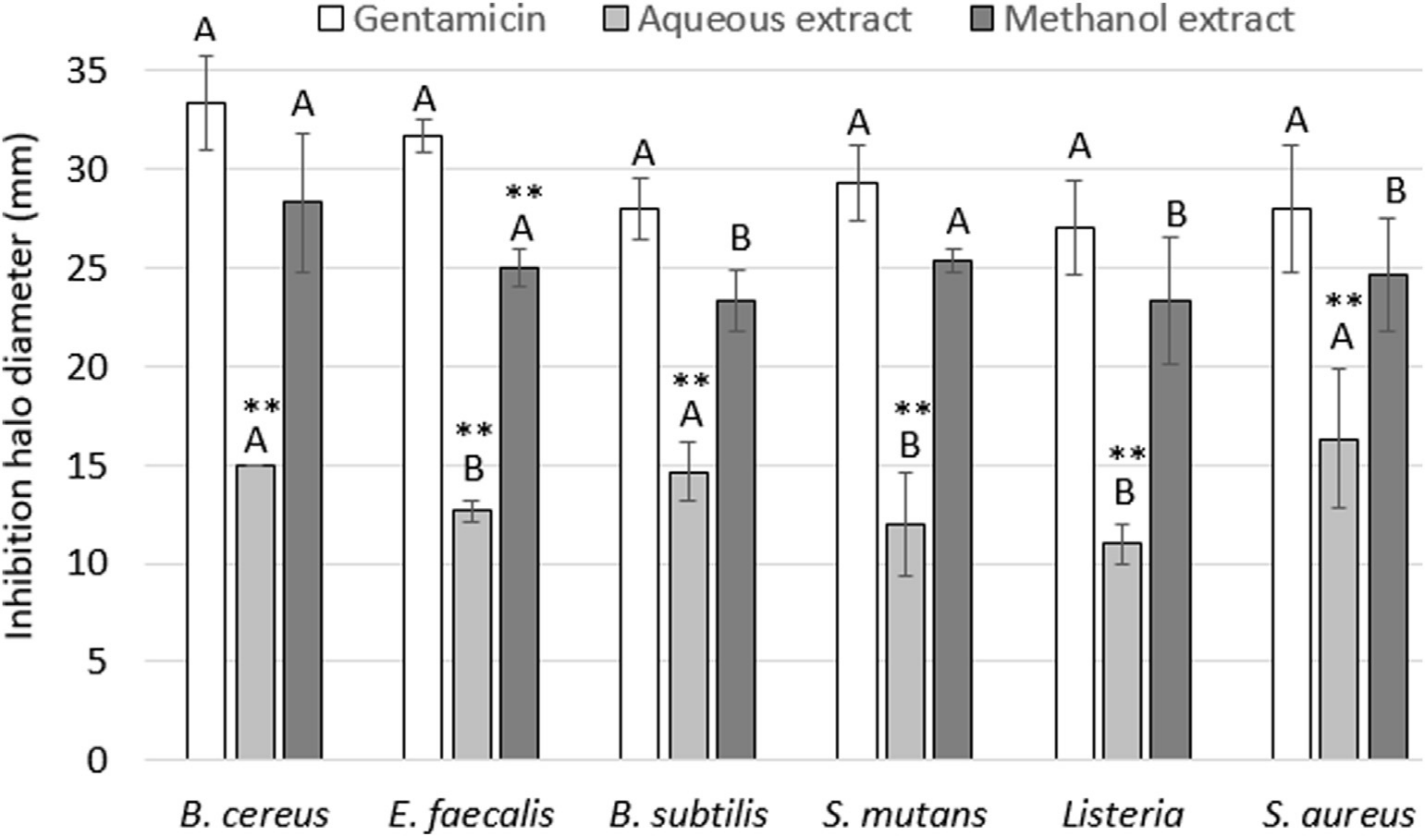
Diameter of the inhibition halo generated by the methanol and aqueous extracts of *Jacaranda mimosifolia* flowers against gram-positive microorganisms. The results express the average of four independent experiments with three replicates each. Different letters per bar color indicate statistical difference between microorganisms (Tukey Test p > 0.05). ∗∗ Indicates statistical difference with respect to the positive control (Gentamicin) (Dunnet Test p > 0.05).

**Figure 3:**
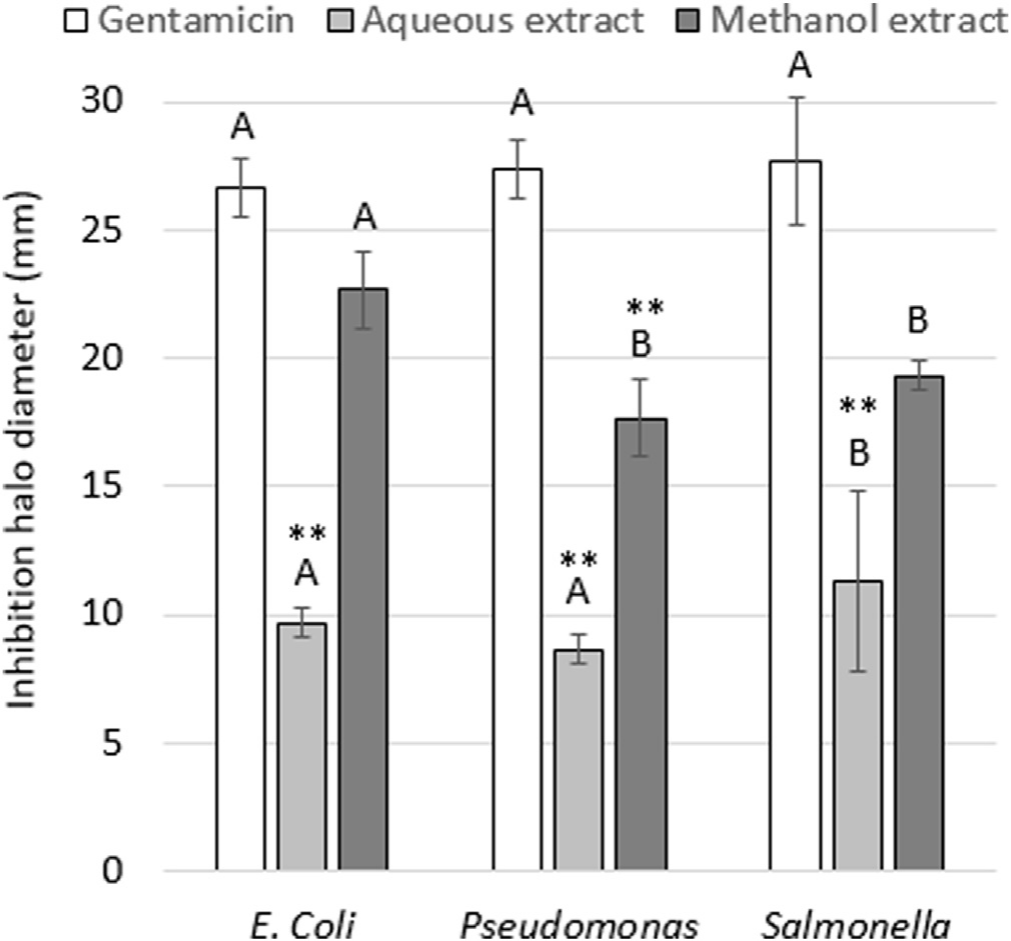
Diameter of the inhibition halo generated by the methanol and aqueous extracts of *Jacaranda mimosifolia* flowers against gram-negative microorganisms. The results express the average of four independent experiments with three replicates each. Different letters per bar color indicate statistical difference between microorganisms (Tukey Test p > 0.05). ∗∗ Indicates statistical difference with respect to the positive control (Gentamicin) (Dunnet Test p > 0.05).

Figure 2 and Figure 3 show the diameter of the inhibition halos generated by the methanol and aqueous extracts of *Jacaranda mimosifolia* flowers against gram-positive and gram-negative microorganisms, respectively. The positive control (Gentamicin) was taken as a reference to determine the antimicrobial activity, presenting a diameter of the inhibition halo ranging from 26 to 33 mm depending on each of the evaluated microorganisms. Gentamicin is a broad-spectrum aminoglycoside antibiotic which action mechanism is through active transport, crossing the cellular membrane of susceptible bacteria and irreversibly binding to the 30S ribosomatic subunits, preventing the start of protein synthesis, and inducing cell death (Yoshizawa et al., 1998).

The methanol extract showed greater antimicrobial activity with a range of 17.66–28.33 mm of inhibition halo, compared to the aqueous extract with a range of 8.66–16.33 mm) for all the evaluated microorganisms. On the other hand, the gram-positive microorganisms presented a larger diameter of the inhibition halo compared to the gram-negative for both methanol and aqueous extracts. The most susceptible gram-negative bacterias were *E. coli* (22.66 ± 1.52 mm) for methanol and *S. typhimurium* (11.33 ± 3.51 mm) for aqueous extracts. These results show that *Jacaranda mimosifolia* extracts could be a potential resource to avoid resistance to antibiotics, a recurrent health problem, principally in gastrointestinal diseases caused by toxins of these bacteria. For gram-positive, the most susceptible were *B. ceureus* (28.33 ± 3.51 mm) for methanol and *S. aureus* (16.33 ± 3.51 mm) for aqueous extracts. Species of the genus *Jacaranda* are part of the folklore and ethnobotanical practices in certain areas of South America. The flowers of this tree are used as medicinal treatments and have been attributed with anti-inflammatory, anti-parasitic, anti-fungal, and anti-cancer properties (Hussain et al., 2007), while other species in this genus have been used to treat both viral and bacterial infections (Gachet and Schühly, 2009). Pereira et al. (2015) reported an inhibition halo of 96.9% and 96.6% on *Bacillus cereus* and *S. aureus,* respectively, using an ethanol extract of leaves of *J. oxyphylla*. Zatta et al. (2009) evaluated the antibacterial activity of *Jacaranda decurrens* leaves against 25 strains of *Pseudomonas aeruginosa*, an opportunistic pathogen that causes respiratory tract infections, showing significant inhibitory activity. On the other hand, *J. mimosifolia* is part of the traditional medicine in Ecuador, used to purify the blood and treat venereal diseases (Acosta-Solís, 1992). Hendra et al. (2018) evaluated nine metabolites isolated from *J. mimosifolia* flowers, including phenylethanoid β-D-glucopyranose, a new compound, which did not presented inhibitory activity against on *S. aureus, E. coli, Klebsiella penumoniae*, *P. aeruginosa*, *Acinetobacter baumannii*, *Candida albicans* and *Cryptococcus neoformans* at a low concentration (32 μg/ml). Furthermore, the results of the present work coincide with the results of Rojas et al. (2006), which reported inhibitory activity against *B. cereus* and *E. coli* for aqueous extract of *J. mimosifolia* leaves.

**Figure 4:**
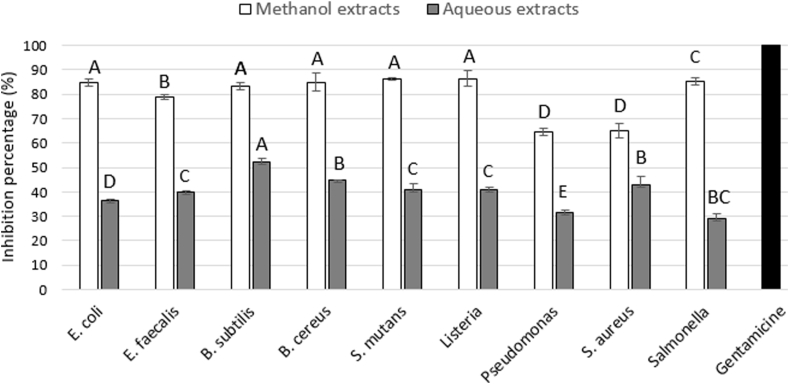
Inhibition percentage of the methanol and aqueous extracts of *Jacaranda mimosifolia* flowers against microorganisms of interest. White bars indicate percentage inhibition for methanol extract and gray bars correspond to the aqueous extract. Gentamicin was used as a positive control (black bar), presenting a minimum or null bacterial growth. Negative controls (methanol and water) showed no inhibition halos. The results express the average of three independent experiments with three replicates each. Different letters per bar color indicate statistical difference between microorganisms (Tukey Test p > 0.05).

Figure 4 shows the inhibition percentages of bacterial growth. The methanol extracts presented higher percentages compared to the aqueous extracts. The results showed the following susceptibility of the microorganisms against the methanol extract of *J. mimosifolia* flower; *Listeria = S. mutans > B. cereus = E. coli > B. subtilis > E. faecalis > Salmonella > S. aureus > Pseudomonas*, presenting inhibition percentages ranging from 86.4 to 64.6%. In contrast, lower ranges were observed for the aqueous extract of *J. mimosifolia* flower (52.3%–31.7%), with the following trend; *B. subtilis > B. cereus > S.aureus > Salmonella = S. mutans > Listeria > E. faecalis > E. coli > Pseudomonas*.

The results of the methanolic extract of *J. mimosifolia* flower are relevant since the percentage of inhibition on *Listeria monocytogenes* and *Streptoccus mutans* (86.4% in both cases) qualifies it as an alternative of natural antimicrobial to prevalent health problems worldwide. Several studies on the antibacterial activity of plant extracts and plant parts on *Listeria monocytogenes* have been performed; this pathogen is responsible for listeriosis, a disease with a high mortality rate (20%) in risk populations and considered a major food safety problem worldwide (Pouillot et al., 2012; Ceruso et al., 2020). Some of these investigations attribute the inhibitory effect on *L. monocytogenes* to the presence of phytochemical compounds, such as alkaloids, polyphenolic compounds, gallic acid, glycosides, tannic acid, flavonoids and sterols (Batool et al., 2018). Following this line, in the present work, 86.4% of inhibition for *Streptococcus mutans* was found with the methanol extract of the *Jacaranda* flower. This information is significant since *S. mutans* is present in the multifactorial process that originates dental caries, and this might be the first assay of *J. mimosifolia* extracts on this microorganism as no previous reports were found. The high concentration of flavonoid-type compounds reported in this work could be responsible for the inhibitory effect on these types of microorganisms. Gutiérrez-Venegas et al. (2019) tested various polyhydroflavones against a group of microorganisms related to the dental caries process where bacteria and fungi were included by measuring the inhibition diameter; in all cases, a bacteriostatic effect was observed on the evaluated bacteria and fungi. In recent years, research related to the formation of anti-cariogenic biofilms has been carried out using polyphenolic compounds, mainly flavonoids, observing an inhibitory effect on the adhesion of hydroapatite pellets treated with proanthocyanidins exposed to *Streptococcus,* resulting in a decrease of hydrophobicity in bacteria depending on the concentration of various flavonoids, increase of membrane permeability of bacteria, as well as decrease of acids produced by *S. mutans* in biofilms with the flavonoid apigenin (Koo et al., 2002, 2003).

In particular, part of the antimicrobial activity has been attributed to the phenolic compounds present in the extracts from plants. Studies on the chemical components presented in *J. mimosifolia* indicate the presence of triterpenes, quinones, fatty acids, flavonoids, and acetosides, a novel phenylethanoid dimmer (Moharram and Marzouk, 2007). Li et al. (2017) indicated that the most common compounds that act as antimicrobials include the members of the flavonoids family which are activated and/or synthesized in the presence of biotic and abiotic stress. On the other hand, Mori et al. (1987) showed that the presence of hydroxyl groups in 3 ′, 4′ and 5′of the B ring and in C3 influences the antimicrobial activity of flavonoids. Data corroborated by Zheng et al. (1996) demonstrated a relationship between the structure and antibacterial activity against gram-positive bacteria using methylated flavones, and that the most active were those with hydroxyl groups at C-5 and C-7 and three substitutions at ring B.

## 4 Conclusion

*Jacaranda mimosifolia* flowers represent a source of polyphenolic compounds, showing that methanol and aqueous extracts have high antioxidant activity. Furthermore, both extracts showed antibacterial activity against gram-positive and gram-negative pathogens, with a predominance over gram-positive bacteria. The antioxidant and antibacterial results obtained in this work raise the possibility of using and revaluing *Jacaranda* flowers as a source of compounds of interest to the pharmaceutical, food, medical, cosmetic, and agricultural sectors. The findings of the present study justify deeper research on the applications of the *J. mimosifolia* flower as an antimicrobial of natural origin.

## Declarations

### Author contribution statement

A.A. Feregrino-Perez: Conceived and designed the experiments; Analyzed and interpreted the data; Contributed reagents, materials, analysis tools or data; Wrote the paper.

H. Aguirre-Becerra: Analyzed and interpreted the data; Wrote the paper.

S.A. Pineda-Nieto: Conceived and designed the experiments; Performed the experiments; Analyzed and interpreted the data.

B.L. Álvarez-Mayorga: Conceived and designed the experiments; Contributed reagents, materials, analysis tools or data.

D.M.R. Pastrana: Conceived and designed the experiments; Analyzed and interpreted the data; Contributed reagents, materials, analysis tools or data.

J.F. García-Trejo and R.G. Guevara-González: Contributed reagents, materials, analysis tools or data.

### Funding statement

A.A. Feregrino-Perez was supported by FOFI 2018.

### Data availability statement

Data included in article/supplementary material/referenced in article.

### Declaration of interests statement

The authors declare no conflict of interest.

### Additional information

No additional information is available for this paper.
